# First Report of Bacterial Kidney Disease (BKD) Caused by *Renibacterium salmoninarum* in Chum Salmon (*Oncorhynchus keta*) Farmed in South Korea

**DOI:** 10.3390/microorganisms12112329

**Published:** 2024-11-15

**Authors:** Kyoung-Hui Kong, In-Ha Gong, Sung-Ju Jung, Myung-Joo Oh, Myung-Hwa Jung, Hyun-Ja Han, Hyoung Jun Kim, Wi-Sik Kim

**Affiliations:** 1 Department of Aqualife Medicine, Chonnam National University, Yeosu 59626, Republic of Korea; kongkh@korea.kr (K.-H.K.); inha90ng@gmail.com (I.-H.G.); sungju@jnu.ac.kr (S.-J.J.); ohmj@jnu.ac.kr (M.-J.O.); 2 Pathology Division, National Institute of Fisheries Science (NIFS), Busan 46083, Republic of Korea; hjhan77@korea.kr; 3 Department of Aqualife Medicine, Kongju National University, Yesan 32588, Republic of Korea; mhjung@kongju.ac.kr; 4 WOAH Reference Laboratory for VHS, National Institute of Fisheries Science, Busan 46083, Republic of Korea

**Keywords:** bacterial kidney disease, histopathology, mariculture, *Renibacterium salmoninarum*, 16S rRNA gene

## Abstract

In 2021, a prominent increase in mortality was observed in juvenile and subadult cultured chum salmon (*Oncorhynchus keta*) on a mariculture farm in Jeollanam-do Province, South Korea. The affected fish displayed distinct symptoms: pale gills, petechial hemorrhages in the muscles, and white nodules on the kidneys. Infectious pancreatic necrosis virus (IPNV) was cultured from some fish samples using fish cell lines. Bacteria were isolated from various fish tissues using kidney disease medium-two (KDM-2) culture medium. By detecting and sequencing the 16S rRNA gene using DNA extracted from the kidneys of the infected fish via PCR, the isolated bacteria were identified as *Renibacterium salmoninarum*. Histopathological examination primarily focused on hematopoietic tissues of kidneys and revealed clear evidence of severe necrosis and granulomatous changes. Additionally, nuclei with peripherally displaced chromatin were abundant in the kidneys of affected fish. These findings suggest that mass mortality of chum salmon was caused by *R. salmoninarum*, which induced typical bacterial kidney disease (BKD) symptoms, without IPNV infection. This represents the first outbreak of BKD attributed to *R. salmoninarum* infection in farmed chum salmon in South Korea.

## 1. Introduction

In 2021, cultured juvenile and subadult chum salmon (*Oncorhynchus keta*) from migrating wild chum salmon spawners underwent mass mortality in a marine farm in Jeollanam-do Province, Korea. This incident raises concerns about the health of farmed populations, particularly in light of the first report of bacterial kidney disease (BKD) caused by *Renibacterium salmoninarum* in farmed chum salmon in South Korea.

Chum salmon is widely distributed in the North Pacific Ocean, with approximately 76% of them caught in Korean, Japanese, and Russian waters over the last 20 years [1]. The main part of their distribution is Russia and northern Japan, with relatively small populations inhabiting the southern range of its distribution off the Korean Peninsula [1]. In Korea, a chum salmon enhancement program was initiated in 1913 [2]. The initial activities involved capturing adult individuals for artificial fertilization and fry release. Between 2016 and 2020, approximately 340,000 adult fish were caught and 58,257,000 offspring were released in Korean waters [3].

In Korea, chum salmon are not a focus fish species for mariculture production; rather, they are bred to release their offspring and boost local population sizes. However, recent efforts have been made to promote the mariculture of salmonids to increase salmon production. Salmonid species offer several advantages, including (i) high expected demand due to their popularity as a protein source in Korea, (ii) the potential to offset the overproduction of olive flounder (*Paralichthys olivaceus*), a main mariculture species, (iii) suitability for winter cultivation in empty farms in southern coastal areas, and (iv) the potential industrial value of polydeoxyribonucleotides extracted from salmonid fish sperm, which are known to promote tissue regeneration under various pathophysiological conditions [4,5].

Infectious hematopoietic necrosis virus (IHNV), infectious pancreatic necrosis virus (IPNV), and infectious salmon anemia virus (ISAV) were all detected in wild salmonids [6,7,8,9,10,11,12,13]. Sea lice [14,15] and *Gyrodactylus salaris* [16] are the primary parasites causing problems in salmonid industries globally. Among them, IHNV and IPNV were isolated from migrating chum salmon spawners and their offspring in 2006–2008 in Korea [17], although parasitic diseases infecting salmonids never occurred in Korea.

Bacterial kidney disease (BKD) is prevalent among wild and cultured salmonids caused by infection with the Gram-positive bacterium *R*. *salmoninarum* and was first reported in 1930 in Atlantic salmon (*Salmo salar*) in Scotland [18,19]. BKD can lead to significant mortality in juvenile salmonids in both fresh- and seawater environments as well as in pre-spawning adults [20]. Over the last nine decades, BKD has been commonly observed in cultured salmonid populations across North and South America, continental Europe, Japan, Scandinavia, and other regions [19,20]. Infections caused by *R. salmoninarum* generally progress slowly, with noticeable symptoms rarely appearing until the fish are 6–12 months old [21]. Fish severely affected by *R. salmoninarum* infections may exhibit no external signs or may display various symptoms, such as lethargy, darkened skin, exophthalmos, abdominal distension due to ascites, pale gills, petechial hemorrhages on the body, and grayish-white nodular lesions in the kidney, and sometimes in the spleen and liver [9,18,20,21,22]. Currently, no reports related to *R. salmoninarum* isolation or detection have been published for any fish species in Korea, including salmonids.

In 2021, an outbreak of disease at an aquaculture farm in Goheung on the south coast of Korea caused a high mortality rate (50 to 100%) in cultured juvenile and subadult chum salmon. Mortality in subadult fish was first observed during seawater adaptation and continued for a total of 89 d, with 100% cumulative mortality. In contrast, the death of juvenile fish began at 66 d after the outbreak of disease in subadult fish and continued for a total of 260 d, with 50% cumulative mortality. Most diseased fish showed similar clinical signs with severe petechial hemorrhages in the lateral muscles and grayish-white nodules in the kidneys. So far, BKD and its causative agent, *R*. *salmoninarum*, have not been detected in any fish species including salmonids in Korea. Therefore, the authors of this study estimated that this disease was IHN. However, IHNV was not detected in the diseased fish.

Here, we conducted diagnostic tests on moribund fish using bacteriological, parasitological, virological, and histological examinations to identify the parasite and/or pathogens responsible. The identification of the causal organism(s) is the first step in providing clear recommendations to limit chum salmon mortality in the future.

## 2. Materials and Methods

### 2.1. Diseased Fish and Clinical Signs

**Figure 1:**
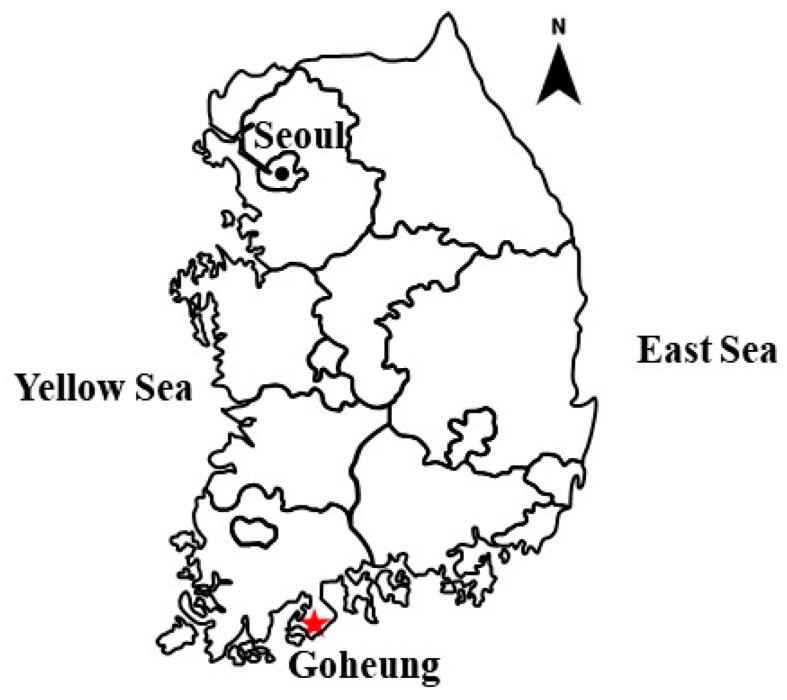
Geographical location of the aquaculture farm in Goheung, Korea.

**Figure 2:**
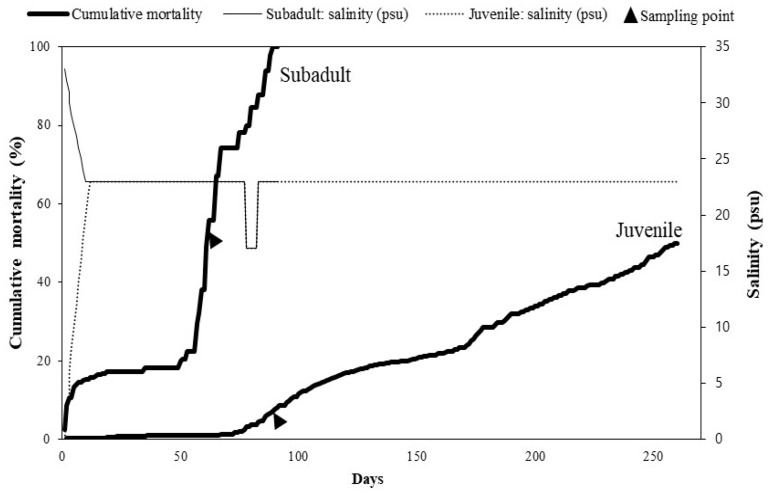
Cumulative mortality of subadult and juvenile chum salmon relative to changes in salinity.

In 2021, 16,000 juvenile and 280 subadult-stage chum salmon were reared in a land-based marine farm located in Goheung on the south coast of Korea (Figure 1). The juvenile fish (mean weight: 2.9 ± 0.1 g) were initially kept in underground freshwater at 15 °C before gradually transferring them to seawater at a rate of 2–3 psu/day using a gradual adaptation method in square tanks (9 × 9 × 1 m) (Figure 2). Conversely, the subadult fish (289 ± 50 g) were first acclimated to seawater (33 psu) at 15 °C before gradually transferring them to underground freshwater at a rate of 1 psu/day in square tanks (9 × 9 × 1 m) (Figure 2). After acclimating both juvenile and subadult fish at 23 psu, the tanks were connected to a recirculation aquaculture system at temperatures of 15–17 °C and 23 psu using seawater and underground freshwater. Approximately 10–30% water at 23 psu was added daily to the recirculation system.

### 2.2. Diagnostic Examinations for the Affected Fish

Moribund and dead fish were removed from the chum salmon population. Twelve moribund fish, i.e., six juvenile fish (mean ± standard deviation = 40 ± 14 g) and six subadult fish (429 ± 108 g), were collected at 63 and 83 d, respectively. Fish were transported on ice and immediately subjected to parasitological, bacteriological, and virological examinations. The gills and mucus from skins of the affected chum salmon were examined directly under light (Olympus CX31RBSF, Tokyo, Japan) and phase-contrast (Olympus MVX10) microscopes to identify possible parasitic infections. To isolate bacteria, samples of the kidney, spleen, liver, and nodules in the kidney were cultured on both brain heart infusion agar (BHIA) (BD Difco, Franklin Lake, NJ, USA) and kidney disease medium-two (KDM-2) [23], which were incubated at 15 °C for 7 d and 4 weeks, respectively. The kidneys and spleens were aseptically collected, homogenized in 10 volumes of Hank’s balanced salt solution (HBSS, Gibco, Waltham, MA, USA), and centrifuged at 3000× *g* for 30 min. The supernatant was filtered through a 450 nm membrane filter, and 100 µL of filtrates were inoculated onto fathead minnow (FHM, ATCC), chinook salmon embryo (CHSE-214, provided by Prof. Yoshimizu Mamoru), and epithelioma papulosum cyprini (EPC, ATCC) cells in a 24-well tissue culture plate (Nunc, Roskilde, Denmark). The inoculated cells were then incubated at 15 °C for 14 d and observed daily for evidence of cytopathic effects (CPEs).

### 2.3. Polymerase Chain Reaction (PCR), Reverse Transcriptase (RT)-PCR, and Sequencing Analysis

PCR for two fish bacterial pathogens (*R. salmoninarum* and *Piscirickettsia salmonis*) and RT-PCR for six fish viral pathogens (IHNV, IPNV, ISAV, salmonid alphavirus (SAV), viral hemorrhagic septicemia virus (VHSV), and hirame novirhabdovirus (HIRRV)) were performed using previously reported methods. The primers utilized for pathogen gene detection via PCR are presented in Table 1.

DNA and RNA were extracted from 50 mg of kidney and spleen tissue from each of the 12 moribund fish, bacterial colonies were obtained from KDM-2, and viruses were sourced from virus-culture media from the cell cultures. Each sample was isolated using a DNA extraction kit (Roche, Mannheim, Germany) or TRIZOL reagent (Gibco, USA) following the manufacturer’s instructions. Reverse transcription (RT) was performed using the TOYOBO ReverTra Ace qPCR RT kit (Toyobo, Osaka, Japan) according to the manufacturer’s instructions. Synthesized cDNA or DNA was amplified using 12 PCR primer sets for the eight pathogens (Table 1). Additionally, PCR was performed with 27F (5′-AGAGTTTGATCMTGGCTCAG-3′) and 1492R (5′-TACGGYTACCTTGTTACGACTT-3′) primers targeting 16S rRNA [33] to identify any bacteria obtained from KDM-2. The PCR product was loaded onto 1.5% agarose gel and purified using a QIAquick gel extraction kit (Qiagen, Hilden, Germany). The amplified products were subjected to nucleotide sequence analysis using an ABI PRISM 3730 XL DNA analyzer (Applied Biosystems, Foster City, CA, USA) according to the manufacturer’s instructions. The resulting sequences were assembled using the program MEGA11 [34].

### 2.4. Histopathology and Immunohistochemistry (IHC)

The kidneys were removed from four live moribund juvenile fish and immediately fixed in 10% neutral buffered formalin for 48 h. Thereafter, standard histological procedures were performed for tissue dehydration and paraffin embedding. Tissue sections with a thickness of 5 μm were stained with hematoxylin and eosin (H&E) and examined using a light microscope. To detect *R. salmoninarum*, IHC was performed using a standard protocol [35,36,37]. Briefly, endogenous peroxidase activity was blocked by incubating slides with 3% H_2_O_2_ for 15 min at room temperature. After washing with phosphate-buffered saline (PBS) for 5 min, non-specific protein binding sites were blocked by incubation with 10% normal goat serum (Abcam, Cambridge, UK) at room temperature for 1 h. Following the blocking step, the slides were incubated with an anti-*R. salmoninarum* monoclonal antibody (1:10 dilution in PBS) (Aquatic Diagnostic Ltd., Scotland, UK) for 1 h and washed four times with PBS for 5 min each. The slides were subsequently treated with a 1:1000 dilution of goat anti-mouse IgG conjugated with horseradish peroxidase (HRP) (GWVitek, Seoul, Republic of Korea) for 1 h. Colorimetric detection was performed using a 3.3′–diaminobenzidine (DAB) substrate kit (Abcam, UK) following the manufacturer’s instructions. After color development, the reaction was stopped by adding distilled water. Subsequently, the cells were counterstained with Mayer’s hematoxylin (Sigma-Aldrich, St Louis, MO, USA) and mounted using the Histo-Choice Mounting Medium (Ameresco, Framingham, MA, USA).

## 3. Results and Discussion

**Figure 3:**
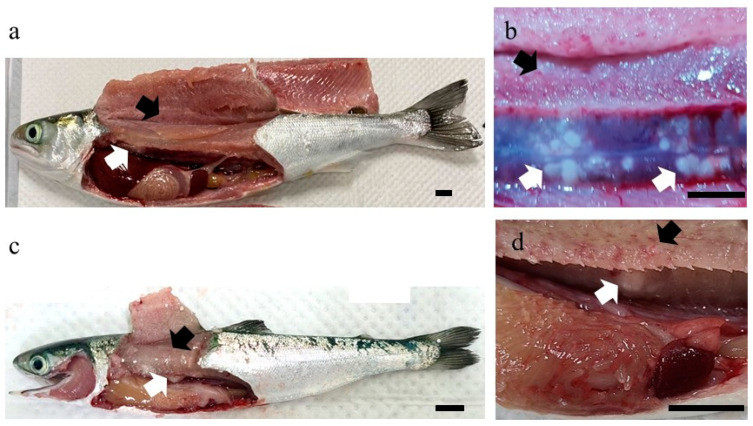
Affected chum salmon, showing petechial hemorrhages in the muscle (black arrowhead) and grayish-white nodules in the kidney (white arrowhead) of (**a**,**b**) subadult and (**c**,**d**) juvenile fish. Scale bars = 1 cm.

During mariculture of chum salmon in an aquaculture farm located in South Korea in 2021, high mortalities occurred in juvenile and subadult fish. In subadult chum salmon, fish mortality was first recorded during the freshwater adaptation (salinity: 33 psu) period, and this trend persisted for 10 d, resulting in a cumulative mortality of 15%. Furthermore, the mortality was sustained below 23% until 56 d post-transportation, with the calculated mortality rates ranged from 1 to 6.5% per day (Figure 2). At 57 days post-transportation, mortality recurred and 100% (280/280 fish) cumulative mortality was observed at 89 d. In juvenile chum salmon, fish deaths occurred 66 d after seawater transportation, leading to a cumulative mortality of 50% (8000 out of 16,000 fish) by day 260 (Figure 2). The most affected fish exhibited lethargy and swam slowly near the water surface. All the six subadult fish subjected to testing exhibited severe petechial hemorrhages in the lateral muscles and grayish-white nodules in the kidneys (Figure 3a,b). Among these, three individuals also showed pale gills. Similarly, all six affected juvenile fish that were sampled displayed the same lesions as those in the subadult fish, and three of them exhibited enlarged spleens (Figure 3c,d).

**Figure 4:**
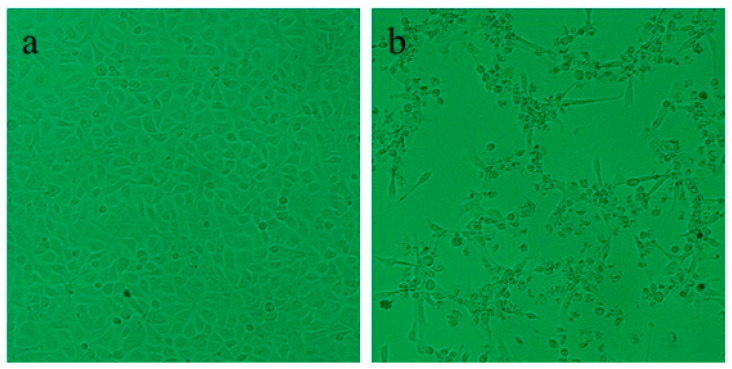
Morphology of CHSE-214 cell lines: (**a**) normal CHSE-214 cell (×100) and (**b**) CHSE-214 infected with IPNV (×100).

In microscopic observation to confirm parasitic infection, no parasites were found on the gills or body surfaces of the affected fish. In the virological examination of juvenile fish, 2 of 12 samples of kidney and spleen tissue homogenates displayed CPEs in CHSE-214 cells 6 d post-inoculation. CPEs were characterized by cell rounding and subsequent cell lysis (Figure 4). PCR analysis of the cell cultures showing CPEs was performed for virus identification (Table 2). Specifically, the two samples tested positive for IPNV, whereas PCR amplification targeting VHSV, HIRRV, or IHNV, which are notorious pathogenic viruses in Korean fish farms, tested negative (Table 2). Both nucleotide sequences were found to be identical to each other and showed 97.9% homology with the China IPNV isolate (ChRtm213, GenBank number: KX234591, # 1493, 1565: C→T; # 1529, 1553, 1594: T→C; # 1673: A→G; # 1674: C→A) taken from rainbow trout. These nucleotide sequences also shared 97.35% identity with that of the Korean DRT isolate of IPNV (GenBank number: D26526), taken from rainbow trout. Among the twelve subadult chum salmon sampled, no viral pathogens such as IHNV, IPNV, ISAV, SAV, VHSV, and HIRRV were detected (Table 2).

**Figure 5:**
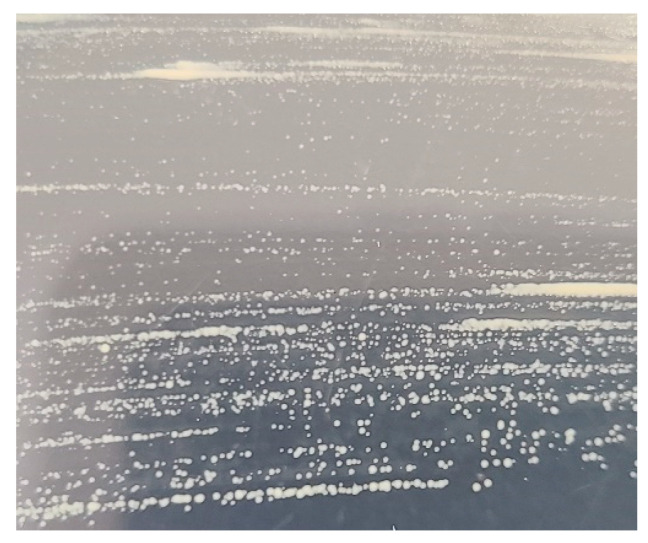
Appearance of *Renibacterium salmoninarum* colonies on KDM-2 after incubation for 19 d at 15 °C.

**Figure 6:**
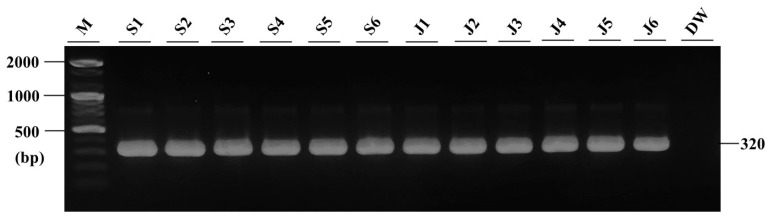
PCR result using DNA extracted from 12 diseased fish (M = marker; S1–S6 = subadult fish; J1–J6 = juvenile fish; DW = negative control).

Regarding the gross signs displayed by diseased fish, severe petechial hemorrhages were observed in the muscle tissue, along with grayish-white nodules in the kidneys of both juvenile and subadult fish. These clinical signs closely resemble those of typical BKD in salmonids, as reported in previous studies [9,18,20,21]. Furthermore, no bacteria were isolated from the kidney, spleen, liver, and kidney nodules on BHIA. However, numerous identical colonies were isolated from the kidney, spleen, liver, and kidney nodules of all diseased fish (six juveniles and six subadults) on the 19th day of incubation with KDM-2 (Figure 5). The colonies were characterized by a creamy white, round, and shiny appearance and did not exceed 2 mm in diameter. Upon analyzing the 16S rRNA gene sequences of the two bacteria identified from the diseased fish by KDM-2, the nucleotide sequences were found to be identical to each other and exhibited 100% homology with *R. salmoninarum* isolated from coho salmon (GenBank number: MT023376). DNA extracted from spleen and kidney tissue of diseased fish was used to detect *R*. *salmoninarum* via an *R*. *salmoninarum*-specific primer set (Table 2). The amplified PCR product was loaded on agarose gel (Figure 6). However, no specific bacterial gene, specifically *P. salmonis*, was detected in any of the 12 fish, suggesting that mortality was affected by *R. salmoninarum* infection.

**Figure 7:**
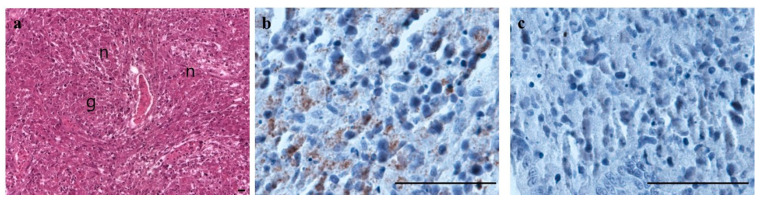
Histopathology and immunohistochemical analysis of kidney tissues of chum salmon. (**a**) Histopathology of the kidney showing tissue necrosis and granuloma by hematoxylin and eosin staining. g: necrosis in the central part of a granuloma; n: necrotic cells. Scale bars = 100 μm. Immunohistochemical analysis showing a positive reaction of necrotic cells in the kidney (**b**) and no reaction in the negative control (**c**). Bar = 50 μm.

Histological examination revealed signs of necrosis and granulomatous reaction in the kidney tissues. The most frequent microscopic finding was diffuse necrosis in the hematopoietic tissues of all the tested fish (Figure 7a). Granulomatous reactions were observed in one of the four fish (Figure 7a). Epithelial cell necrosis in the tubules and infiltration of cell debris into the lumen of the renal tubules were observed in all the tested fish. Histological sections of affected kidneys revealed the presence of necrosis and granulomatous reactions, similar to those commonly found in BKD in salmonid fish [9,22,38]. Immunohistochemistry revealed a positive reaction in the necrotic tissue and within the cytoplasm of the kidney (Figure 7b), and a positive intracellular reaction against bacteria was observed. Numerous *R. salmoninarum*, which appear dark yellow with IHC staining, were prominently distributed in the blue background of the kidney tissues. In contrast, the negative control (lacking a primary antibody) exhibited no reaction, resulting in no dark-yellow staining (Figure 7c). Based on the histopathological observations, we could not recognize the typical signs of IPNV infection. Therefore, we verified that the specific mortalities occurring in both subadults and juveniles were related to *R. salmoninarum* infection. Additionally, the bacterial infection in juvenile fish was suggested to be influenced by diseased subadults infected with *R. salmoninarum* occurring in the same recirculation aquaculture system.

Several studies indicated the efficacy of antibiotics such as erythromycin, azithromycin, and enrofloxacin in BKD treatment [39,40,41]. To control the disease, erythromycin was orally administered daily to juvenile chum salmon from days 98 to 147 (at 100 mg/kg body weight) and from 165 days to one year (at 200 mg/kg body weight). Despite these efforts, the mortality rate did not decrease, eventually resulting in a cumulative mortality of 100% in the juvenile chum salmon over the course of the year. BKD is one of the most challenging bacterial diseases to control in fish [19]. While various antibiotics, including erythromycin, were reported to effectively treat BKD, they do not entirely eliminate the pathogen, creating the risk for antibiotic-resistant strains to emerge [19,39,40,41].

In Korea, *R. salmoninarum* was not previously isolated or detected in any fish species prior to the commencement of our study. BKD is not included in the diseases listed by the World Organization for Animal Health (WOAH) or the Aquatic Life Disease Control Act of Korea. Consequently, legal regulations and disease control methods for BKD are not yet established in Korea, amplifying its potential to cause significant damage to the mariculture farms of the country. Furthermore, during the BKD outbreak, *R. salmoninarum* continued to spread from farms to the marine environment. In Korea, species such as olive flounder and rockfish (*Sebastes schlegelii*) are cultured in marine environments. Thus, assessing the susceptibility of these fish species through challenge tests is urgently required.

The juvenile and subadult chum salmon in this study originated from the seed of wild chum salmon that returned to the Korean Peninsula. According to Suzuki et al. [42], *R. salmoninarum* was detected in the ovarian fluid of chum salmon returning to Hokkaido, Japan, at rates ranging from 0 to 98%. *R. salmoninarum* transmission occurs both horizontally through cohabitation (as demonstrated in our study) and vertically through direct transfer from ovarian tissues during ovulation [21,25,43,44,45]. Prior to this study, *R. salmoninarum* was neither isolated nor detected in any of the fish species in Korea. This suggests that *R. salmoninarum* likely originated from wild chum salmon returning to the region. Therefore, a follow-up survey of *R. salmoninarum* in wild chum salmon returning to the Korean Peninsula and other salmonids inhabiting outbreak areas should be conducted in the near future.

## 4. Conclusions

This report marks the first outbreak of bacterial kidney disease caused by *R. salmoninarum* in cultured chum salmon from a marine environment in Korea. Furthermore, the pathogen was successfully cultured in KDM-2 medium, and its identity was confirmed through 16S rRNA gene analysis. Our study highlights the importance of early and rapid detection of pathogens that could potentially impact mariculture farms and natural marine environments, so that timely interventions can be proposed to prevent their establishment and spread.

## Figures and Tables

**Table 1:** List of primers used for RT-PCR and PCR detection of viruses and bacteria in kidney tissues of chum salmon.

	^a^ Target Pathogen	Primer Name	Primer Sequence (5′–3′)	Product Length (bp)	Reference
Virus	IHNV	mid-G 1F	AGAGATCCCTACACCAGAGAC	693	[24]
		mid-G 1R	GGTGGTGTTGTTTCCGTGCAA		
	IPNV	P1	AGAGATCACTGACTTCACAAGTGAC	359	[25]
		P2	TGTGCACCACAGGAAAGATGACTC		
	ISAV (segment 7)	Forward	CAGGGTTGTATCCATGGTTGAAATG-	155	[26]
		Reverse	GTCCAGCCCTAAGCTCAACTC		
	ISAV (segment 8)	Forward	CTACACAGCAGGATGCAGATGT	104	
		Reverse	CAGGATGCCGGAAGTCGAT		
	ISAV [segment 6 (HPR)]	Forward	GACCAGACAAGCTTAGGTAACACAGA	304	
		Reverse	GATGGTGGAATTCTACCTCTAGACTTGTA		
	SAV	E2F	CCGTTGCGGCCACACTGGATG	516	[27]
		E2R	CCTCATAGGTGATCGACGGCAG		
	VHSV	3F	GGGACAGGAATGACCATGAT	319	[28] [29]
		2R	TCTGTCACCTTGATCCCCTCCAG		
	HIRRV	oPVP278	ACTACAATCAACAAATCGCA	730	[30]
		oPVP279	GTTGGCGAGTGGGATGTTG		
Bacteria	*Piscirickettsia salmonis*	EubB (27F)	AGAGTTTGATCMTGGCTCAG	1540	[31]
		EuBA (1518R)	AAGGAGGTGATCCANCCRCA		
		PS2S (223F)	CTAGGAGATGAGCCCGCGTTG	467	
		PS2AS (690R)	GCTACACCTGAAATTCCACTT		
	*R*. *salmoninarum*	P3	AGCTTCGCAAGGTGAAGGG	383	[20] [32]
		M21	GCAACAGGTTTATTTGCCGGG		
		P4	ATTCTTCCACTTCAACAGTACAAGG	320	
		M38	CATTATCGTTACACCCGAAACC		

^a^ IHNV, infectious hematopoietic necrosis virus; IPNV, infectious pancreatic necrosis virus; ISAV, infectious salmon anemia virus; SAV, salmonid alphavirus; VHSV, viral hemorrhagic septicemia virus; HIRRV, hirame novirhabdovirus.

**Table 2:** Results of virus isolation in cell culture and specific gene detection of fish pathogens from chum salmon.

**Group**	**Fish**		**Appearance of Cytopathic Effect in Cell Line (%)** **(No. Positive/No. Tested Samples)**			**Detection by RT-PCR Rate (%)** **(No. Positive/No. Tested Samples)**						**Detection by PCR Rate (%)** **(No. Positive/No. Tested Samples)**		
	**Tested** **Sample**	**Mean** **Weight (g)**	**CHSE-214**	**EPC**	**FHM**	**HIRRV**	**IHNV**	**IPNV**	**ISAV**	**SAV**	**VHSV**	***R*** **. *salmoninarium***		***P*** **. *salmonis***
												**Nested PCR**	**16S rRNA**	**Nested PCR**
**Subadult**	6	429 ± 108	0% (0/6)	0% (0/6)	0% (0/6)	0% (0/6)	0% (0/6)	0% (0/6)	0% (0/6)	0% (0/6)	0% (0/6)	100% (6/6)	100% (2/2)	0% (0/6)
**Juvenile**	6	40 ± 14	33% (2/6)	0% (0/6)	0% (0/6)	0% (0/6)	0% (0/6)	0% (0/6)	0% (0/6)	0% (0/6)	0% (0/6)	100% (6/6)	100% (3/3)	0% (0/6)
						0% (0/2 ^a^)	0% (0/2 ^a^)	100% (2/2 ^a^)	0% (0/2 ^a^)	0% (0/2 ^a^)	0% (0/2 ^a^)	NT	NT	0% (0/2 ^a^)

RT-PCR, reverse transcription polymerase chain reaction; HIRRV, hirame novirhabdovirus; IHNV, infectious hematopoietic necrosis virus; IPNV, infectious pancreatic necrosis virus; ISAV, infectious salmon anemia virus; SAV, salmonid alphavirus; VHSV, viral hemorrhagic septicemia virus; *R*. *salmoninarium*, *Renibacterium salmoninarum*; *P*. *salmonis*, *Piscirickettsia salmonis*; NT, not tested. ^a^ Virus culture media of samples showing CPE in CHSE-214 cell.

## Data Availability

Data are contained within this article.
